# Characterization of Biocalcium Microparticles from Saltwater Crocodile (*Crocodylus porosus*) Bone and Their Potential for Enhancing Fish Bologna Quality

**DOI:** 10.3390/foods14101732

**Published:** 2025-05-13

**Authors:** Theeraphol Senphan, Natthapong Mungmueang, Supatra Karnjanapratum, Sutee Wangtueai, Akkasit Jongjareonrak, Suthasinee Yarnpakdee

**Affiliations:** 1 Program in Food Science and Technology, Faculty of Engineering and Agro-Industry, Maejo University, Sansai, Chiang Mai 50290, Thailand; theeraphol_s@mju.ac.th (T.S.); maxnatthaphong_2541@hotmail.com (N.M.); 2 Faculty of Agro-Industry, Chiang Mai University, Chiang Mai 50100, Thailand; supatra.ka@cmu.ac.th (S.K.); sutee.w@cmu.ac.th (S.W.); jakkasit@gmail.com (A.J.); 3 Cluster of Innovation for Sustainable Seafood Industry and Value Chain Management, Chiang Mai University, Chiang Mai 50100, Thailand

**Keywords:** biocalcium, fish bologna, saltwater crocodile bone, functional gel enhancer

## Abstract

Saltwater crocodile (SC; *Crocodylus porosus*) bone, an underutilized by-product, can be converted into high-value bio-calcium (Biocal), serving as a potential source of calcium and minerals. This study aimed to produce SC bone Biocal as functional gel enhancer for fish bologna development and to increase calcium intake. The resulting bone powder was evaluated for physicochemical, microbiological, and molecular properties. Additionally, the textural, physicochemical, structural, and sensorial properties of the formulated fish bologna incorporating Biocal at varying levels (0–10% *w*/*w*) were also evaluated. Biocal, obtained as a fine white powder, had a 16.83% yield. Mineral analysis showed 26.25% calcium and 13.72% phosphorus, with no harmful metals or pathogens detected. X-ray diffraction confirmed hydroxyapatite with 69.92% crystallinity, while calcium bioavailability was measured at 22.30%. Amino acid analysis indicated high levels of glycine, proline, and hydroxyproline, essential for collagen support. The findings confirmed that SC bone Biocal is beneficial and safe for food fortification. Incorporating SC Biocal (2–10% *w*/*w*) significantly affected the fish bologna characteristics (*p* < 0.05). As the Biocal level increased, the gel strength, hardness, and shear force also increased. The addition of 6% (*w*/*w*) Biocal significantly improved the textural property, without a detrimental effect on the sensory attributes of the bologna gel (*p* < 0.05). SDS-PAGE analysis showed TGase-enhanced myosin heavy chain (MHC) cross-linking, particularly in combination with Biocal. Moreover, the enriched Biocal–bologna gel exhibited a finer and denser microstructure. Thus, SC Biocal, particularly at 6% (*w*/*w*), can serve as a functional gel enhancer in surimi-based products, without compromising organoleptic quality.

## 1. Introduction

Calcium is vital for human health, especially bone and teeth [1]. Because of inadequate consumption or poor intestinal absorption, calcium deficiency is a global issue that is associated with osteoporosis and decreased bone mass. Calcium-fortified products are a strategy for improving calcium intake. Commercial calcium, especially tricalcium phosphate (TCP) and calcium carbonate (CaCO_3_) have been widely used to supplement for calcium inadequacies [2]. However, inorganic calciums are often poorly absorbed in the intestine due to low solubility. Bio-calcium (Biocal) is a natural source of calcium associated with peptides that can be prepared from animal bones. It has an advantage in terms of absorption over calcium alone, suggesting that animal bone can serve as a promising source of dietary calcium [3,4]. Jung and Kim [5] found that Biocal derived from fish bone contained a high level of phosphopeptides, which increased the proportion of soluble calcium salts, particularly within the gastrointestinal tract, thereby enhancing calcium absorption. In addition, Biocal derived from animal bone is typically found in the form of calcium hydroxyapatite (HA; Ca_10_(PO_4_)_6_(OH)_2_), as detected in human bones. Recently, Biocal powders from aquamarine bone have been successfully produced and characterized [4,6], with different sources and processing methods influencing their properties.

Saltwater crocodiles (SC; *Crocodylus porosus*) are primarily farmed in Thailand for their leather, while their meat is utilized as a food material. In 2021, Thailand exported crocodile-derived products valued between USD 600,000 and 750,000 [7]. After the slaughtering process, the leftover bones are often discarded as waste. However, these bones contained a wide range of nutritional components, including calcium, amino acids, chondroitin, and collagen, which can be converted into functional ingredients [8]. To address environmental concerns and promote full utilization, these bones are being explored as a promising source for Biocal production, due to their high calcium content and unique structural properties [9]. Aquaculture and marine bone have been effectively used to produce Biocal and use it in many fortified foods such as crackers, cookies, sausages, surimi gel, and mayonnaise [4]. However, information regarding the extraction and characterization of Biocal from crocodile bone has rarely been reported.

With the growing demand for functional food products, there is increasing interest in dietary calcium that enhances nutritional value and promotes health benefits [10]. Salmon (*Salmo salar*) belly, the offcut obtained from filleting, is rich in important bioactive components and bioavailable nutrients including high-quality protein, necessary long chain omega-3 fatty acids, minerals, and essential vitamins like B 12 and D [11]. These nutrients contribute to cardiovascular health and overall well-being. Additionally, salmon offers a unique taste, making it a versatile ingredient for various palatable dishes.

Fish bologna is one of the most popular emulsion-type sausages, made from a mixture of surimi, animal fat, and water. It is widely consumed worldwide for its unique textural properties. However, the raw materials of surimi contain low levels of omega-3 polyunsaturated fatty acids (ω-3 PUFA) due to lipid removal during washing. The fatty fish meat, particularly salmon protein, has a lower gel-forming ability than derived from ordinary fish meat. To enhance PUFA levels in formulated bologna, omega-3 fatty fish have gained attention as a supplement to white-meat surimi, despite the lower gel-forming capacity of salmon protein. Gel strength, a key property of gelation, serves as an indicator of surimi-based gel quality. The textural characteristic depends on myofibrillar protein. In general, calcium fortification is widely accepted in surimi-based products, to increase nutritional value and improve gelling properties. A sufficient calcium amount is essential to activate endogenous transglutaminase (TGase), varying by fish species. Moreover, a stronger gel can be achieved through calcium bridges that promoted protein cross-linking. Therefore, this research aimed to produce Biocal from SC bones as a nutritional gel enhancer in fish bologna development. The effect of incorporation at different levels (0–10%) on the physicochemical, textural, and sensory characteristic of fortified bologna made from white meat surimi blended with salmon belly strip in the presence of microbial TGase (MTGase) was investigated. This research not only highlights the potential of SC bones as a source of Biocal but also explores innovative approaches to enhance the quality of surimi-based products.

## 2. Materials and Methods

### 2.1. Materials and Chemicals

Frozen threadfin bream (*Nemipterus virgatus*) surimi (Grade AA) was purchased from Anusorn Mahachai Surimi Co., Ltd. (Muang, Samut Sakorn, Thailand). The surimi consisted of fish flesh, refined sugar, and polyphosphate at 93.80%, 6.0%, and 0.2% (*w*/*w*), respectively. Raw SC (*Crocodylus porosus*) bone, sourced from Uni-Croc Co., Ltd. (Wang Thonglang, Bangkok, Thailand) was minced and packaged in 2 kg portions in polyethylene bags, then stored in a foam box. The surimi was transported to the Faculty of Agro-Industry at Chiang Mai University, while the bone was delivered to the Pilot Plant of the Food Science and Technology Program at Maejo University (Chiang Mai, Thailand) via a temperature-controlled trailer (−20 °C) within 24 h. Upon arrival, the frozen samples were stored at −20 °C for no more than one month. Ingredients for bologna preparation, including sugar, salt, tapioca flour, shortening, spices, and an orange coloring agent, were purchased from a supermarket in Chiang Mai Province, Thailand

All chemicals used in the experiments were of analytical grade and were obtained from Union Science Trading (Chiang Mai, Thailand). Food-grade MTGase was supplied by Tinnakorn Chemical & Supply (Pra Khanong, Bangkok, Thailand).

### 2.2. Preparation and Characterization of SC Biocal

#### 2.2.1. Preparation of SC Biocal with Different Treatments

Frozen minced SC bone was processed to create Biocal using a protocol with the following steps:

(1) High-pressure heating process: Frozen minced SC bone samples were thawed under running water for about 1 h until they reached a temperature of 20 °C. Subsequently, the samples were autoclaved using an autoclave (Model No. 1925x All American, Miami, FL, USA) at 121 °C and 15 psi for 20 min.
(2) Alkaline-soaked process: Autoclaved bone was subjected to high-pressure heating in a 10:1 (*v*/*w*) ratio and immersed in a 1.0 M KOH solution at 70 °C for 30 min while being stirred at 500 rpm with an overhead stirrer (Scilogex Os20-s LED Digital Overhead Stirrer, Pacific Star Corporation, Houston, TX, USA). Following drainage of the KOH solution, the treated bone was rinsed with running water at ten times the original volume for 5 min, with continuous stirring until the rinse water had a neutral pH. The resulting bones were then dried at 50 °C for 2 h in a cabinet tray dryer (Binder FED115, Tuttlingen, Germany) and then ground to a size of 3–4 mm.
(3) Ethanol immersion process: The alkaline-soaked bone was immersed in 95% (*v*/*v*) ethanol at a ratio of 1:10 (*w*/*v*) at 25 °C and stirred for 30 min. Following this, the ethanol was drained, and the bone was washed with three volumes of ethanol for an additional 30 min. After stirring and draining, the resulting product was subjected to a bleaching and grinding step.
(4) Bleaching and grinding process: The ethanol-immersed bone was soaked in a 2% (*v*/*v*) hydrogen peroxide (H_2_O_2_) solution at a bone-to-H_2_O_2_ ratio of 1:10 (*w*/*v*) at room temperature for 30 min, while being continuously stirred. After treatment, the sample was rinsed with ten volumes of running water. Subsequently, the sample was dried in a cabinet tray dryer at 50 °C for 5 h. Finally, the dried samples were crushed into a powder using a pin mill (Bonny YPT-302S, Yor Yong Hah Heng Ltd., Bangkok, Thailand), resulting in a product known as Biocal powder. The resulting Biocal was subjected to characterization.

#### 2.2.2. Yield of Biocal

The yield was determined and represented as a percentage of Biocal using the following Equation (1):

(1) $$Yield\;(\%)=\left[\frac{weight\;of\;Biocal\;(g)}{weight\;of\;rawbone\;(g)}\right]\times 100$$

#### 2.2.3. Color Measurement

The Biocal samples treated with different treatments were assessed using a colorimeter (ColorFlex EZ, Hunter Lab, Reston, VA, USA) according to the CIE Lab* system. Measurements for L* (lightness), a* (red/green), and b* (yellow/blue) were recorded.

#### 2.2.4. Water Activity (a_w_)

The a_w_ of a 2 g of Biocal sample was determined using a Novasina Lab Master Water Activity Meter (Model aw SPRINT-TH 300, Novasina AG, Lachen, Switzerland), with the sample placed in a plastic cup prior to measurement.

#### 2.2.5. Chemical Composition

Proximate composition: The Biocal subjected to various treatments was analyzed for its chemical composition. This analysis involved measuring the percentage of moisture content, ash, protein, and fat, following AOAC methods 927.05, 942.05, 920.38, and 984.13, respectively [12].

Mineral composition: Biocal sample analysis was performed using a Perkin Elmer Optima 8000 ICP-OES spectrometer (Perkin Elmer, Shelton, MA, USA), following the protocol by Feist and Mikula [13]. The wavelengths for identifying calcium (Ca), phosphorus (P), mercury (Hg), arsenic (As), lead (Pb), and Cadmium (Cd) were 422.7 nm, 213.6 nm, 254.4 nm, 193.7 nm, 220.3 nm, and 228.8 nm, respectively. Data were expressed as percentages based on dry weight.

Hydroxyproline content: The hydroxyproline levels in Biocal samples subjected to various treatments were assessed using the method established by Bergman and Loxley [14]. Biocal powders were treated with hydrochloric acid at 110 °C for 24 h, followed by treatment with activated carbon and filtration. The resulting filtrates were combined with isopropanol, an oxidation solution, and Ehrlich solution, then incubated at 60 °C for 25 min. The absorbance at 558 nm was recorded to quantify the hydroxyproline concentration, using a standard curve ranging from 0 to 60 ppm with the linear regression equation y = 0.1097x − 0.14 and a coefficient of determination (R^2^) of 0.9707. The results were expressed as mg/g of sample.

#### 2.2.6. Bioavailability of Calcium in In Vitro Simulated Gastrointestinal Tract Model System

Calcium bioavailability from the Biocal was assessed using a modified gastrointestinal tract model (GIMs) based on Benjakul et al. [15]. The solubilized calcium after digestion was determination using ICP-OES (Perkin Elmer, Shelton, MA, USA). Bioavailability of calcium percentage was calculated using Equation (2):

(2) $$\mathrm{Bioavailability}\;\mathrm{of}\;\mathrm{calcium}\;(\%)=\left\lceil \frac{Total\;of\;solubilized\;calcium\;content\;after\;digestion}{Total\;of\;calcium\;content\;in\;the\;sample}\right\rceil \times 100$$

#### 2.2.7. Microbial Analysis

Biocal (25 g) was aseptically homogenized with 10 volumes of 0.85% (*w*/*v*) sterile saline using a Stomacher blender (M400, Seward Ltd., Worthing, UK) for 1 min. A series of 10-fold dilutions were then prepared using the same diluent. Total viable count (TVC), yeasts and molds, *Salmonella* spp., *Escherichia coli*, and *Staphylococcus aureus* were assessed following the methodology of Bagade and Patil [16]. Microbial counts were expressed as log CFU/g, except for *E. coli* and Salmonella spp., which were assessed as MPN/g and per 25 g, respectively.

#### 2.2.8. Amino Acid Composition Analysis

The amino acid profiles of Biocal were analyzed using a modified method from Sriket et al. [1]. Samples were hydrolyzed in 4 M methanesulphonic acid with 0.2 mL of 100 M (*v*/*v*) 3-2(2-aminoethyl) indole at 115 °C for 24 h to convert proteins into free amino acids. The hydrolysate was then neutralized with 3.5 M NaOH and diluted with 0.2 M citrate buffer (pH 2.2). A 0.04 mL aliquot was analyzed using an amino acid analyzer (MLC-703; Atto Co., Tokyo, Japan) to quantify the amino acid composition of the Biocal samples.

#### 2.2.9. Visual Appearance and Scanning Electron Microscopy (SEM) Analysis

The visual appearance of Biocal samples was recorded using a digital camera (X-A3, Fujifilm, Tokyo, Japan) to evaluate the uniformity, particle size, and surface characteristics.

Scanning electron microscopy (SEM) was used to visualize the microstructure, as described by Sriket et al. [1]

#### 2.2.10. X-Ray Diffraction (XRD)

Phase composition of the samples was analyzed using X-ray diffraction (XRD) with a Philips X’Pert MPD diffractometer and Cu K-alpha radiation. Scanning was performed at 38 degrees per min with a step size of 0.058 degrees over an angular range of 5.8 to 90.8 degrees, operating at 40 kV and 30 mA. Phase identification used JCPDF files No. 01-086-0740 and 01-084-1998 for Biocal sample. Crystallinity was calculated using Equation (3):

(3) $$\mathrm{Crystallinity}\;(\%)=\left\lceil \frac{A(peeak)}{A(total)}\right\rceil \times 100$$

where A (peak) is the area under crystalline peaks, and A (total) is the area under all peaks.

### 2.3. Study on the Impact of SC Biocal Powder on the Characteristic and Properties of Bologna from Salmon Cuts/Surimi Mixed Gel

#### 2.3.1. Preparation of Fish Meat

Frozen surimi and salmon belly cuts was thawed overnight at 4 °C and then chopped into cubes (1 × 1 × 1 cm). To prepare the fish mixture, the surimi and salmon cuts were mixed with a ratio of 70:30 (*w*/*w*) and kept in a polyethylene bag at 4 °C before use.

#### 2.3.2. Bologna Preparation

The original formulation of bologna emulsion sausage was prepared as detailed by Santana et al. [17] with a slight modification, as denoted in Table 1. Briefly, the chopped fish meat was mixed with salt and ice water in a food processor (Panasonic Model MK-5080M, Petaling Jaya, Malaysia) for 30 s. Thereafter, shortening was added and mixed well at high-speed level for 30 s. Other ingredients, including sugar, spices, carrageenan, MTGase, and tapioca flour, were added and mixed for another 30 s. SC Biocal with various levels, 2, 4, 6, 8, and 10% (*w*/*w* of total weight), replacement with fish meat, was added to the mixture and blended for another 30 s, with a 5 s rest interval, for a total of 4 min. The resulting bologna paste was stuffed into polyvinyl ethylene chloride casings with diameters of 2.5 cm and 7 cm, with both ends sealed tightly. To allow gelation, the bologna mixed gels were incubated at 40 °C for 30 min, followed by heating at 90 °C for 20 min. Subsequently, all gels were cooled in iced water for 30 min and stored at 4 °C overnight prior to analyses. Biocal-fortified bologna samples, at levels of 2, 4, 6, 8, and 10% (*w*/*w*), were referred to as Biocal 2, Biocal 4, Biocal 6, Biocal 8, and Biocal 10, respectively. The control bologna, named Con 1, was prepared in the same manner without the addition of either MTGase or Biocal, while Con 2 included MTGase but no Biocal was added.

#### 2.3.3. Visual Appearance

All cylindrical control and Biocal-fortified bologna mixed gels (2.5 cm in diameter and 2.5 cm in length) and sliced samples (7 cm in diameter and 1 mm in thickness) were photographed using a smartphone camera (iPhone 14, Apple Inc., Cupertino, CA, USA).

#### 2.3.4. Textural Properties

Gel penetration: Bologna gel analysis was carried out using a Model TA-XT2 texture analyzer (Stable Micro Systems, Surrey, UK). Before analysis, gels stored at 4 °C were equilibrated to room temperature (28–30 °C). Cylindrical samples (2.5 cm in length) were prepared and subjected to determination of breaking force (g) and deformation (mm) using a spherical plunger (diameter 5 mm; depression speed 60 mm min^−1^). Gel strength was then calculated as shown in Equation (4):

Gel strength (g × cm) = Breaking force (g) × Deformation (cm)(4)

Textural profile analysis (TPA): TPA was performed using a TA-XT2i texture analyzer (Stable Micro Systems, Surrey, UK) with a cylindrical aluminum probe (35 mm diameter). The samples were cut into cylindrs (2.5 cm height × 2.5 cm diameter) and used for determination of hardness, springiness, cohesiveness, adhesiveness, gumminess, and chewiness.

Shear force: Shear force was measured from cylindrical samples (7 cm diameter, 1 cm height) using a Warner-Bratzler attachment on a texture analyzer, following the method of Migdał et al. [18]. Blade speed during the test was 1.5 mm/s. The maximum force (g) to cut the sample was recorded.

#### 2.3.5. Expressible Moisture Content (EMC)

EMC of Biocal-fortified bologna mixed gels was measured according to the method of Buamard and Benjakul [19]. Biocal- fortified bologna gel samples (a thickness of 5 mm and weight of X g) were positioned between three layers of Whatman No.1 filter paper underneath and two layers on top. After a standard weight (5 kg) was placed on the top of the sample for 2 min, the weight of the sample (Y g) was measured. EMC was calculated as follows (Equation (5)):

Expressible moisture (%) = [(X − Y)/X] × 100(5)

#### 2.3.6. Whiteness

Color value of Biocal-fortified bologna mixed gel was analyzed using Hunterlab (ColorFlex, Hunter Associates Laboratory, Reston, VA, USA). Lightness (L*), redness/greenness (a*), and yellowness/blueness (b*) were measured. Whiteness index was calculated using the subsequent equation (Equation (6)):

Whiteness (%) = 100 − [(100 − L*)^2^ + a*^2^ + b*^2^]^1/2^ (6)

#### 2.3.7. Protein Pattern

Protein patterns of Biocal-fortified bologna mixed gel were analyzed by Sodium Dodecyl Sulfate-Polyacrylamide Gel Electrophoresis (SDS-PAGE) under a reducing condition, according to the method of Laemmli [20]. Samples were solubilized using a 5% SDS solution (90 °C). Protein content (15 μg) was loaded on gel, consisting of 4% stacking gel and 10% separating gel. Electrophoresis was conducted at 15 mA/gel.

#### 2.3.8. Sensory Evaluation

Sensory analysis of Biocal-fortified bologna mixed gel was performed using a 9-point hedonic scale [21]. A likeness evaluation of the bologna gel was carried out by 60 untrained panelists aged 22 to 40 who were familiar with processed fish products. The ready-to-eat bologna was prepared following good hygiene practices. Prior to testing, samples were reheated by steaming at 100 °C for 5 min (ensuring a core temperature of not less than 70 ± 2 °C). Thereafter, the samples were cut into bite-sized pieces (2.5 cm in diameter and 1 cm in thickness), placed in covered white plastic cups, and served at room temperature under daylight-style fluorescence. The panelists were asked to evaluate the texture, color, odor, flavor, taste, sandy mouthfeel, and overall liking of gel samples. Between the samples, the panelists were instructed to rinse their mouths with water. The experiment involving human participants was approved by the Human Research Ethics Committee of Chaing Mai University (CMUREC No. 66/192). The certificate of exemption (COE No. 038/66) was granted on 29 August 2023.

#### 2.3.9. SEM Images

The microstructure of the control and Biocal-fortified bologna gels was examined using scanning electron microscopy (SEM) at an accelerating voltage of 5 kV, following the method outlined by Wijayanti et al. [22]. Gel samples were cut into 1–2 mm cubes and fixed with 2.5% (*v*/*v*) glutaraldehyde in 0.2 M phosphate buffer (pH 7.2) for 3 h at room temperature. After fixation, the samples were rinsed with distilled water and dehydrated in an ethanol series (25%, 50%, 70%, 80%, 90%, and 100%). The dehydrated samples were then subjected to critical point drying (CPD) and coated with gold. SEM imaging was performed at a magnification of 13,000×.

### 2.4. Statistical Analysis

Experiments were conducted in triplicate, and data were analyzed using ANOVA with mean comparisons via Duncan’s multiple range test, as described by Steel et al. [23]. Analyses were carried out with SPSS version 17.0 software (SPSS for Windows, SPSS Inc., Chicago, IL, USA) with a significance level at *p* < 0.05.

## 3. Results and Discussion

### 3.1. Characterization of SC Biocal

#### 3.1.1. Yield, Color, and Water Activity (a_w_)

The yield percentage and color of SC Biocal prepared under different treatments are shown in Table 2. The raw bone exhibited a yield of 100%, serving as the baseline for comparison. Subsequent pretreatments, including high-pressure heating (autoclaving), alkaline soaking, ethanol immersion, and bleaching, resulted in a reduced yield of 16.83 ± 0.29%. Heating pretreatment significantly decreased the yield, likely due to protein denaturation and the removal of organic matter during autoclaving [15]. Alkaline soaking facilitated protein removal, while ethanol immersion targeted lipid extraction. In the final stages, the bleaching and grinding processes further refined the Biocal from the treated bone [1]. The yield of Biocal from SC bone with these treatments was likely diminished due to a combination of thermal degradation, precipitation of calcium, incomplete breakdown of organic matter, and physical losses throughout the processing stages. Therefore, the progressive yield decline across treatments reflects the trade-off between purity and material retention, underscoring the need for optimized processing methods to balance yield and quality in Biocal production [17]. Jindapon et al. [24] reported that a yield of 13.4% was obtained under optimal conditions for extracting hydroxyapatite from Bigeye Snapper (*Priacanthus tayenus*) bone, which were found to be using 5% HCl for 60 min.

The color values of SC Biocal with different treatments are shown in Table 2. The L* value was found to be 90.28 ± 0.02, indicating that the Biocal had a light color. The a* value was −0.32 ± 0.06, suggesting a slight greenish tint, while the b* value was 8.25 ± 0.25, indicating a mild yellow hue. These color parameters suggest that the Biocal had a pale, slightly greenish-yellow appearance, which may have been influenced by the organic components in the bone or the processing methods used during extraction [1]. The final treatment, which involved bleaching and grinding, resulted in the brightest appearance, nearing a white color. Using H_2_O_2_ as a bleaching agent improved the whiteness and decreases yellowness, which can lead to pigment oxidation and degradation. Its decomposition creates oxidizing agents that disrupt chromatophore integrity, while OH- breaks bonds within chromatophores, reducing their light absorption [15]. This treatment effectively minimizes reddish and yellow hues, resulting in a visually appealing product suitable for food and supplement applications. Therefore, each treatment effectively enhanced the brightness of the Biocal and altered its color profile towards a lighter, more neutral product, which is advantageous for applications in the food and dietary supplement industries, where color perception is essential.

The water activity (a_w_) of the SC Biocal was found to be 0.51 ± 0.01. This relatively low water activity indicates that the Biocal product was microbiologically stable and unlikely to support the growth of most bacteria, yeasts, and molds. According to established food microbiology principles, microbial growth is generally inhibited at a_w_ values below 0.60, with most pathogenic bacteria requiring an a_w_ above 0.85 for proliferation [1]. Therefore, the measured a_w_ value of the SC Biocal suggests that it would be highly resistant to microbial spoilage under proper storage conditions. This low a_w_ also contributes to extending the shelf life of the Biocal powder, minimizing the risk of microbial contamination during storage and handling. However, while a low water activity enhances the stability of the product, it is still important to ensure that Biocal is stored in moisture-proof packaging to prevent moisture uptake, which could otherwise increase the a_w_ and potentially allow microbial growth. These findings highlighted the significance of moisture control in maintaining the safety and quality of Biocal products.

#### 3.1.2. Chemical Composition

The proximate composition of Biocal extracted from SC bone is presented in Table 2. The moisture content was found to be 4.87 ± 0.06%, indicating a relatively low amount of water present in the final product. The protein content was 10.38 ± 0.32%, suggesting that the bone material retained a moderate level of protein after extraction. The lipid content was 2.80 ± 0.09%, reflecting a low-fat content in the Biocal, which is typical for calcium-based products. The ash content, which is primarily composed of minerals, was 69.71 ± 0.14%, indicating a high level of mineral content, likely calcium, in the Biocal. These results suggest that the Biocal extracted from SC bone was predominantly composed of minerals, with a relatively small proportion of proteins and lipids. The alkaline soaking treatment, aimed at protein removal, resulted in a substantial reduction in moisture, with protein levels decreasing significantly and the lipid content dropping markedly, indicating the effective extraction of protein and reduction in unwanted fats [25]. Ethanol immersion further decreased both lipid and moisture contents, while maintaining protein levels, confirming its efficiency in lipid removal without excessive loss of protein. Finally, the bleaching and grinding process resulted in the lowest moisture and lipid content, while significantly increasing the ash content, suggesting a high mineral content in the final Biocal product [1]. Sriket et al. [1] reported that Biocal production from hybrid catfish bones using the alkaline soaking process resulted in an ash content increase from 35.34% to 68.15%, indicating a higher mineral content. This cleaning likely removed residual meat and lipids, with solvents and bleaching agents affecting the moisture, protein, lipid, and ash contents. Bone composition varies by species, age, size, and season [26].

The analysis of mineral content in the SC Biocal revealed significant findings relevant to its nutritional and safety profiles. The calcium and phosphorus contents in the Biocal from saltwater crocodile bone were 26.25% and 13.72%, respectively (Table 2). Idowu et al. [27] found that Biocal derived from both alkaline and non-alkaline treated salmon bones contained higher levels of calcium and phosphorus. Additionally, the analysis showed that mercury levels were below 5 µg/kg, indicating minimal contamination and enhancing the safety of the Biocal product. Notably, arsenic, lead, and cadmium were reported as non-detectable, which is crucial for consumer safety, as the absence of these heavy metals further validated the product’s suitability for dietary supplementation. Thus, these results demonstrate that the processing methods applied to the saltwater crocodile bone effectively preserved and enhanced its mineral content, while ensuring safety from harmful contaminants.

Table 2 also shows the hydroxyproline content of the Biocal extracted from SC bone, showing a value of 2.85 ± 0.26 mg/g sample. Hydroxyproline is a key amino acid in collagen and its presence in the Biocal suggests the retention of some organic material, specifically collagen, during the extraction process [28]. The measured hydroxyproline content indicates that the Biocal still contained traces of collagen, which may influence its texture and potential applications. The relatively low level of hydroxyproline further implies that a significant portion of the organic matter, including collagen, may have been removed or degraded during the extraction process, leaving behind a more mineral-rich product [1]. Benjakul et al. [29] reported that the hydroxyproline content in Biocal powders from precooked tongol tuna bone and yellowfin tuna bone was 17.88 mg/g and 15.80 mg/g, respectively.

#### 3.1.3. Ca Bioavailability (%)

Table 2 illustrates the calcium bioavailability of SC Biocal. The results show that the Biocal derived from SCbone displayed a high calcium bioavailability of 16.10 ± 0.37%. The high calcium bioavailability found in the SC bone indicates that it may be an effective source of calcium for use in dietary supplementation. Moreover, Biocal, containing collagen and peptides, releases amino acids during digestion, which lowers pH and enhances the solubility of calcium and phosphorus as free ions, thereby increasing calcium absorption [4]. In the small intestine, these peptides act as carriers to improve absorption [30]. Structural modifications are crucial for influencing Biocal’s composition and performance, significantly affecting its efficacy in gastrointestinal microenvironments (GIMs) [1]. Sriket et al. [31] found that modifications such as cleaning, alkaline treatment, ethanol immersion, bleaching, and grinding enhanced its functional properties and increased calcium bioavailability. Additionally, these findings highlight the importance of selecting appropriate sources for Biocal extraction to optimize mineral bioavailability, which is crucial for enhancing the nutritional quality of functional foods and supplements. Further research is warranted to explore the mechanisms underlying the differences in bioavailability and to evaluate the potential health benefits of these Biocal sources in various populations.

#### 3.1.4. Microbial Assessment

The microbial assessment of the SC Biocal, as outlined in Table 2, reflected the hygienic practices employed during the production process. The microbiological analysis showed that the total viable count was not detectable, indicating the absence of microbial contamination and demonstrating the effectiveness of the processing methods used to ensure initial product quality. Additionally, the absence of yeasts and molds further enhanced the microbiological stability of the Biocal, which is essential for preventing spoilage and prolonging its shelf life. Importantly, Salmonella spp. was not detected, significantly reducing the potential health risks associated with pathogenic contamination, while *Escherichia coli* levels were recorded at less than 3 MPN/g, indicating good sanitary quality. Furthermore, undetectable levels of *Staphylococcus aureus* provided additional assurance of consumer safety. Overall, the microbial profile of the SC Biocal suggests that it is a microbiologically safe product at the point of production, offering reassurance to consumers, particularly those vulnerable to foodborne illnesses. Nevertheless, it is important to note that the maintenance of low microbial levels depends on appropriate packaging, storage, and handling practices. Proper storage conditions are crucial for preserving the microbial quality of Biocal during its shelf life [32].

#### 3.1.5. Amino Acid Composition

Table 3 shows that the Biocal contained various amino acids, with glycine being the most abundant at 4.40 g/100 g, followed by proline at 2.95 g/100 g and glutamic acid at 1.67 g/100 g. Other notable amino acids included L-alanine (2.32 g/100 g), L-arginine (1.59 g/100 g), and aspartic acid (0.94 g/100 g). The presence of these amino acids suggests that the Biocal contained a mix of both essential (4.30 g/100 g) and non-essential (17.14 g/100 g) amino acids, which may have implications for its functional properties and potential uses in various applications [1]. The spectrometric method measured 2.85 ± 0.26 mg/g of hydroxyproline (Table 2), indicating a low collagen content in the Biocal. In contrast, the amino acid analyzer detected 2.35 g/100 g, suggesting more collagen was retained. The amino acid analyzer uses HPLC for precise quantification, providing a broader amino acid profile. Proline and hydroxyproline also appeared in substantial amounts, indicating their relevance to collagen stability and connective tissue health [33]. Some amino acids, such as cystine, histidine, and tryptophan, were not detectable (ND). Additionally, the presence of glutamic acid and aspartic acid underscored the nutritional value of this Biocal, suggesting it provides both calcium and a variety of amino acids essential for metabolic processes [34]. Other essential amino acids, such as threonine, lysine, isoleucine, and leucine, were present in lower quantities, indicating that this Biocal could serve as a complementary nutritional source for those lacking these nutrients. Certain amino acids, including cystine, histidine, and tryptophan, were not detected, which may indicate limitations in the amino acid profile. However, the variety of present amino acids still offers potential benefits. Thus, the amino acid composition of Biocal from SC bone highlights its potential as a valuable nutritional supplement, particularly for muscle maintenance and connective tissue health.

#### 3.1.6. Visual Appearance and SEM Microstructure

**Figure 1:**
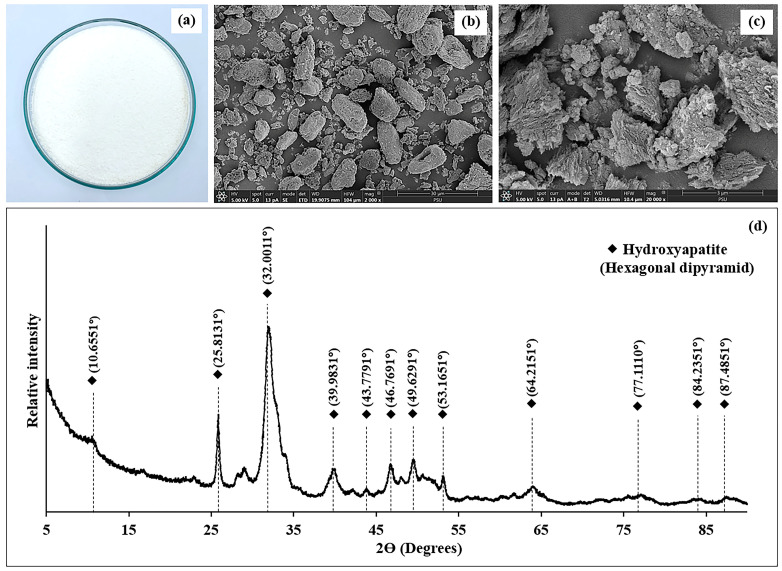
Visual appearance (**a**), SEM microstructure with magnification 2000× (**b**), and 20,000× (**c**), as well as XRD patterns (**d**) of Biocal from SC bone.

The visual appearance of the SC Biocal is displayed in Figure 1a. The SC Biocal appeared as a fine, white powder with a smooth, uniform texture, which is consistent with typical calcium carbonate powders, which are commonly white and free flowing. The fine, uniform texture suggests a high level of purification and grinding, indicating that the Biocal had undergone rigorous processing, such as thermal, alkaline, bleaching, and gridding processes. The absence of visible impurities or discoloration supports the conclusion that the product is clean and well-refined. Its soft and fine consistency is desirable for various applications, including fortification in food products, nutraceuticals, and pharmaceuticals. The fine particle size may enhance the bioavailability of calcium when consumed, facilitating better absorption by the human body. Due to its purity and fine texture, this Biocal could be used in the food industry, especially in calcium-fortified beverages, dairy products, and dietary supplements. Sriket et al. [1] reported that Biocal from hybrid catfish bones was prepared in four steps: high-pressure water jet processing, soaking in alkaline solution, ethanol immersion, and bleaching and grinding to obtain a white micro powder without a fishy odor. Each treatment step not only improved the physical characteristics of the Biocal but also enhanced its potential health benefits, demonstrating the importance of processing methods in the preparation of high-quality calcium supplements.

The SEM analysis of SC Biocal revealed distinct morphological characteristics that highlighted the effects of the various processing treatments. At a magnification of 2000× (Figure 1b), the SEM images exhibited a coarse and irregular structure for the Biocal, indicating the presence of fragmented particles and a rough surface texture that reflected the natural state of the bone material before processing. Upon further magnification to 20,000× (Figure 1c), the images illustrated a more detailed view of the surface morphology, showing a significant reduction in particle size and a smoother texture resulting from the various treatments applied. These structural changes suggest that processing methods, including high-pressure heating, alkaline soaking, and bleaching and grinding, effectively modified the Biocal’s microstructure by enhancing its purity and potentially improving its functional properties. The observed surface characteristics are critical for determining the bioavailability and reactivity of the Biocal for food applications.

#### 3.1.7. X-Ray Diffraction (XRD) Patterns

The XRD analysis of the SC Biocal revealed significant information regarding its crystalline structure and mineral composition (Figure 1d). The diffraction pattern prominently displayed distinct peaks, indicating the presence of hydroxyapatite as the primary crystalline phase, characterized by its hexagonal dipyramidal structure. Notably, the calculated crystallinity of the Biocal was determined to be 69.92%, suggesting a high degree of order in the crystalline lattice of the hydroxyapatite. The presence of sharp diffraction peaks at specific 2θ angles, such as 25.81°, 32.00°, and others, confirmed the crystallinity and purity of the hydroxyapatite phase [35]. These peaks correspond to the specific arrangements of calcium and phosphate ions, which are fundamental to the structural integrity of bone tissue. The intensity of these peaks is indicative of the relative abundance of the crystalline phase, reinforcing the Biocal’s potential as a valuable resource for applications in both nutritional and biomedical fields [36].

The crystallinity of SC Biocal, as shown in Table 2, was found to be 69.92%. This high level of crystallinity suggests a more ordered structure, which enhances the material’s stability and bioavailability. Sriket et al. [1] reported that bio-calcium from hybrid catfish bone had crystallinity levels of 45.19%. Moreover, increased crystallinity is associated with improved solubility and absorption, making it potentially more effective for use in nutritional supplements and food fortification. However, the presence of broader peaks, particularly at lower intensities, may indicate some degree of amorphous content or smaller crystallite sizes. This can influence the material’s properties, such as its reactivity and absorption characteristics, which are critical for its application in health-related products.

### 3.2. The Potential Uses of Biocal in Bologna Made from Salmon Cuts/Surimi Mixed Gel

#### 3.2.1. Textural Properties

##### Gel Penetration

**Figure 2:**
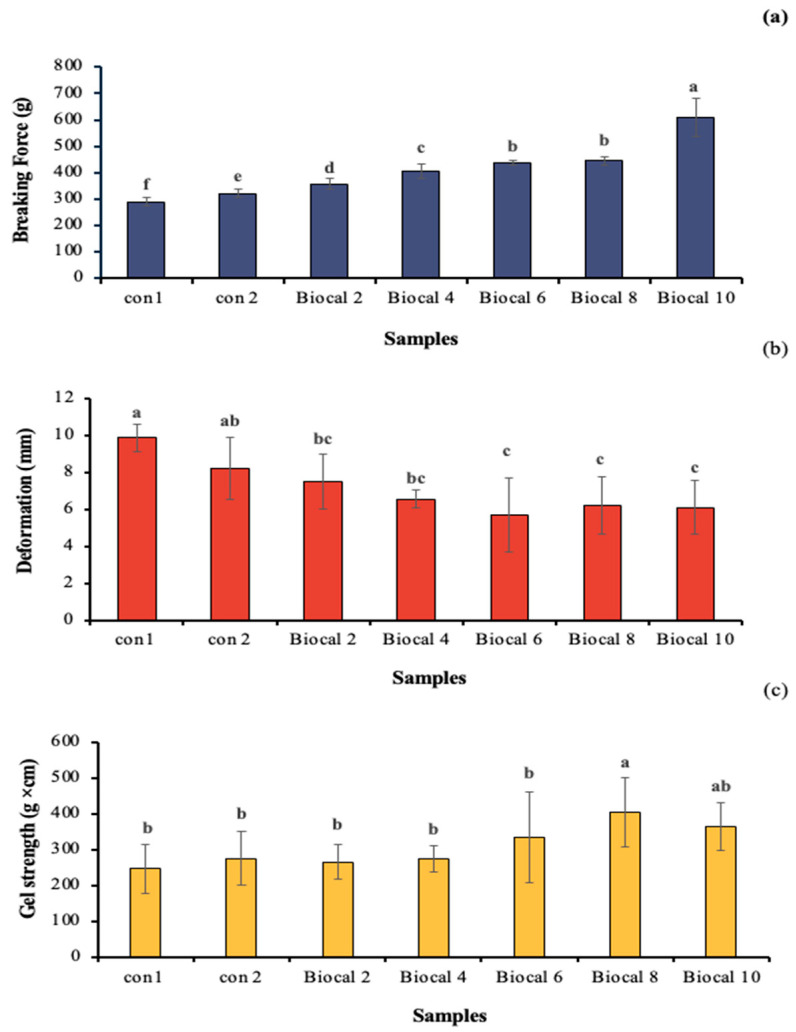
Breaking force (**a**), deformation (**b**), and gel strength (**c**) of bologna made from salmon cuts/surimi mixed gel containing 0.05% MTGase and fortified with SC Biocal at varying levels (0–10%). Different lowercase letters denote significant differences (*p* < 0.05). Values represent mean ± SD (n = 3). Con 1: control bologna gel without MTGase and Biocal; Con 2: control bologna gel containing MTGase without Biocal added; Biocal 2, Biocal 4, Biocal 6, Biocal 8, and Biocal 10: Biocal-fortified bologna gel at 2, 4, 6, 8, and 10% (*w*/*w*), respectively, and incorporated with MTGase.

The gel penetration properties of the Biocal-fortified bologna made from salmon cut/surimi mixed gel were evaluated in terms of breaking force (BF), deformation (DF), and gel strength (GS), as shown in Figure 2. The control gel (Con 1), prepared without MTGase or Biocal, had the lowest BF (290.52 g). When MTGase was applied in the bologna gel (Con 2), the BF of the resulting gel significantly increased, enhancing the gel firmness. MTGase plays a crucial role in forming ε-(γ-glutamyl) lysine linkages during gelation, particularly influencing the setting phenomenon. The addition of Biocal to the bologna mixed gel increased the BF as the Biocal levels rose up to 10%, with which the BF increased by 110%. Biocal provides calcium ions that activate endogenous TGase, promoting cross-linking of myofibrillar proteins to some extent [37]. TGase plays a key role in the formation of isopeptide bonds, specifically ε-(γ-glutamyl) lysine linkages, which are non-disulfide covalent bonds involved in the gel-setting phenomenon. As a result, a stronger gel network is formed in combination with MTGase (calcium-independent enzyme). Furthermore, the calcium liberated from the SC Biocal can form salt linkages with negative charges on the surface of two adjacent proteins [38]. The gel network formation is stabilized by various bonds, thereby increasing gel strength. However, Biocal supplementation diluted myofibrillar protein, especially when a higher level was used. Although the myofibrillar protein was reduced, the Biocal could act as a filler in the gel network, thereby strengthening the gel [22]. However, the DF of the bologna gel tended to decrease as the Biocal levels increased (*p* < 0.05), possibly due to excessive interactions between protein chains, especially in combination with MTGase. Excessive strong bonding in the network could lead to lower elasticity or extensibility. Moreover, Pudtikajorn et al. [39] noted that the reduction in DF of fish tofu fortified with Biocal from scleral cartilage was likely due to the dilution of myofibrillar protein. The dispersion of Biocal might impeded protein crosslinking, especially those formed through hydrogen bonds. For the GS, the value increased from 247 g × cm to 404.89 g × cm when the Biocal levels were increased up to 8% (*w*/*w*). However, a drastic reduction in GS was observed at the maximum Biocal level (10% *w*/*w*). This suggests a dilution effect on the myofibrillar proteins, which are crucial factor for surimi gelation. Although the myofibrillar protein content was reduced, Biocal at an appropriate level contributed to the gel strengthening, especially synergized with MTGase.

##### TPA and Shear Force

The TPA and shear force of bologna mixed gel from salmon cuts/surimi fortified with SC Biocal at varying levels (0–10% *w*/*w*) are shown in Table 4, compared to control gels (Con 1 and Con 2). The hardness and shear force of the Biocal-fortified bologna gel generally increased when increasing the level of Biocal (*p* < 0.05). The gel containing 10% Biocal exhibited the greatest hardness and shear force, measured at 9,051.2 N and 9,168.43 g, respectively. The result was related to the BF and GS, confirming that the Biocal had a gel-strengthening effect on the bologna. The Biocal acted as a filler or binder in the surimi gel, thus generating a better gel network. Additionally, the liberated calcium from Biocal could boost the formation of non-disulfide bonds between proteins by endogenous TGase, particularly with MTGase. The springiness, adhesiveness, and cohesiveness of the Biocal-fortified bologna increased in a dose-dependent manner (*p* < 0.05). Adhesiveness is defined as the work required to overcome the attractive force between a food’s surface and any other contacting material, while springiness shows the elasticity of a food product. Nishinari et al. [40] explained cohesiveness as the force of the inside bonds governing the structure of a product, indicating the food’s capacity to recovery after deformation. The cohesiveness of the Biocal-fortified sample was higher than that of the controls (Con 1 and Con 2), possibly due to the formation of stronger bonds between protein chains through non-disulfide covalent bonds by calcium-activated endogenous TGase in the presence of MTGase. This result suggests that the Biocal preserved the hardness and elasticity of the bologna gels obtained from mixed fish when used at the appropriate level. A similar result was observed for the gumminess and chewiness. Gumminess reflects both the high viscosity and stickiness of a material, whereas chewiness is the amount of force needed to masticate [41]. The results were in line with Wijayanti et al. [42], who reported that the use of Biocal from fish bone improved the textural properties of surimi gel, especially when used alone or in combination with CaCl_2_. Yin et al. [43] found that incorporating micronized fish bone could enhance the qualities of silver carp surimi gel, particularly when the particle size was reduced. Among all treatments, the bologna produced with 8% Biocal (Biocal 8) exhibited the best effect on the textural properties.

#### 3.2.2. EMC, Visual Appearance, and Color

The EMC of the bologna made from salmon cuts/surimi gel and fortified with Biocal at varying levels (0–10%) in the presence of MTGase are shown in Table 4. Typically, EMC represents the water holding capacity (WHC) of surimi gel. A lower content indicates more water retained or bound with gel matrices. The control gels demonstrated higher EMCs than the Biocal-fortified samples (*p* < 0.05). For the bologna without Biocal, Con 2 had a lower EMC than Con 1, suggesting an increased water holding capacity with MTGase. This result was concomitant with the higher in BF, hardness, and shear force. When Biocal was fortified, a decrease in EMC was observed with increasing Biocal levels (*p* < 0.05), possibly because water bound to functional groups in the Biocal and was retained within the dense three-dimensional protein network [44]. At Biocal levels above 4%, the EMC remained relatively stable (*p* > 0.05). Thus, the Biocal in conjunction with MTGase enhanced the crosslinking protein in an ordered fashion and entrapped more water.

**Figure 3:**
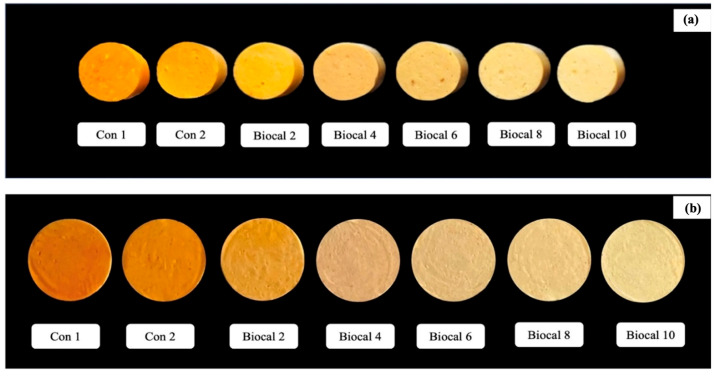
Visual appearances of bologna gel (**a**) and slice (**b**) from salmon cuts/surimi added with SC Biocal at different levels (0–10%). Con 1: control bologna gel without MTGase and Biocal; con 2: control bologna gel containing 0.05% MTGase without Biocal added; Biocal 2, Biocal 4, Biocal 6, Biocal 8, and Biocal 10: Biocal-fortified bologna gel at 2, 4, 6, 8, and 10% (*w*/*w*), respectively, and incorporated with MTGase.

The visual appearance and color of the bologna containing Biocal at different levels (0–10%) are presented in Figure 3 and Table 4, respectively. Biocal fortification at various levels significantly affected the color of the bologna (*p* < 0.05). The bologna samples generally showed an orange-pink color, influenced by an orange coloring agent intended to simulate the color of salmon. With increasing levels of Biocal, the orange color gradually faded, resulting in a paler appearance. The findings were consistent with the observed increase in the whiteness index (*p* < 0.05). This was more likely attributed to the naturally milky white color of the Biocal derived from SC bone. During Biocal preparation, H_2_O_2_ acts as an oxidizing agent to bleach the treated bone, enhancing its whiteness. This bleaching effect might also carry over to the final bologna product when Biocal is used as an additive. The highest L* (lightness), a* (redness), and b* (yellowness) were observed in Biocal 10. The increase in lightness was likely due to light scattering caused by Biocal particles, especially at higher levels. Enhanced cross-linking promoted water retention within the gel structure, leading to greater light scattering. Meanwhile, the elevated a* and b* values may be associated with the Maillard reaction. In this reaction, carbonyl groups generated by lipid oxidation can interact with amino groups in proteins, potentially contributing to the yellowish in the bologna. Wijayanti et al. [22] reported that lipid oxidation occurred during Biocal preparation, as indicated by increasing TBARS. The addition of higher Biocal levels may have introduced more oxidation products, which could have facilitated Maillard reactions, affecting the color of the bologna. Pudtikajorn et al. [39] found that Biocal fortification impacted the color in fish-based gel products. This might be governed by Biocal quality and the type of protein mixture.

#### 3.2.3. Protein Pattern

**Figure 4:**
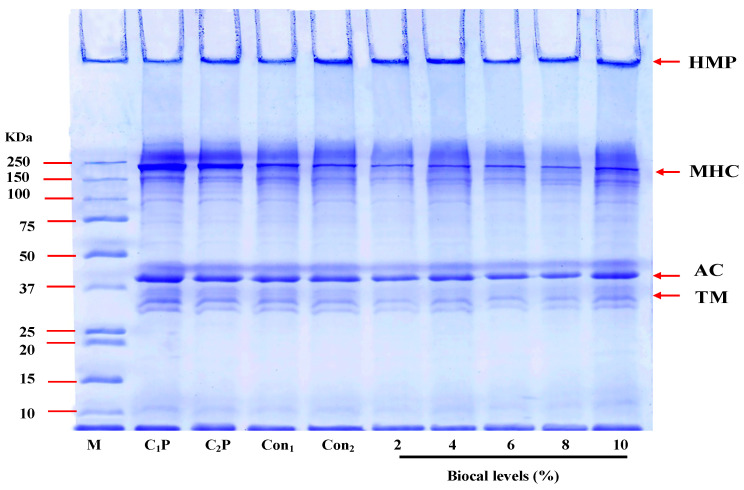
Protein pattern of bologna made from salmon cuts/surimi mixed gel containing 0.05% MTGase and fortified with SC Biocal at different levels (0–10% *w*/*w*). HMP: high molecular weight protein; MHC: myosin heavy chain; AC: actin; TM: tropomyosin M: standard protein marker; C_1_P: control bologna paste without MTGase and Biocal; C_2_P: control bologna paste containing MTGase. Numbers designate the amounts of Biocal added (%).

Figure 4 presents the protein patterns of the bologna gels made from salmon cut/surimi, with and without the addition of SC Biocal at different concentrations (0–10% *w*/*w*) in the presence of MTGase. Myosin heavy chain (MHC) and actin were identified as the major proteins in the bologna pastes (C_1_P and C_2_P). A decrease in MHC band intensity was observed in all gel samples, indicating that MHC polymerization occurred, primarily through non-disulfide covalent bonds. In the control gel (without MTGase and Biocal), the reduction in MHC band intensity indicated the formation of ε-(γ-glutamyl) lysine intra- and inter-molecular cross-links, induced by endogenous TGase. In the presence of MTGase, the reduction in MHC band intensity was more pronounced in the mixed bologna gel, indicating increased protein cross-linking via ε-(γ-glutamyl) lysine isopeptide bonds, as shown by the appearance of high-molecular-weight protein bands in the stacking gel. These cross-links were not dissociated by SDS and β-mercaptoethanol during electrophoresis [45]. TGase contributes to the setting of surimi, where non-disulfide covalent bonds are formed. The formation of isopeptide bonds during setting is associated with increased textural properties, as reflected by the increases in BF and GS observed (Figure 2). Furthermore, no significant changes were observed in actin band intensity. When the Biocal was combined with MTGase, a noticeable decrease in MHC band intensity was observed compared to the control gels. This suggested that the Biocal and MTGase synergistically induced MHC cross-linking, forming cross-linked products. However, no obvious differences in protein pattern were observed with Biocal supplementation at 2–10%. In general, the endogenous TGase from fish muscle is Ca^2+^-dependent enzyme, while MTGase is Ca^2+^-independent and has relatively high acyl transferase activity, which can effectively crosslink myosin molecules [46]. It seems that the Biocal at a level of 2% was sufficient for activation of endogenous TGase. However, gel strengthening was observed when Biocal was added (*p* < 0.05). The results suggested that most cross-linking of this gel was more likely mediated by weak bonds such as H-bond or hydrophobic interaction, which could be destroyed by the SDS used for solubilization and electrophoresis. Thus, the MHC was still crosslinked together by MTGase in the presence of Biocal, indicating that the Biocal had no adverse effect on protein polymerization during heat-induced gelation.

#### 3.2.4. Sensory Evaluation

The sensory scores of the Biocal-fortified bologna made from salmon cuts/surimi mixed gel at varying levels (0–10%) in the presence of MTGase are demonstrated in Table 4. Bologna is a type of sausage that is highly perishable, with a typical pH range of 6.0 to 6.5, which is close to neutral. Its water activity (a_w_) generally falls between 0.96 and 0.98, indicating a high potential for microbial growth and spoilage. To ensure microbial stability, several preservation strategies are employed, including the addition of salt, spices, or antimicrobial agents. Generally, the salt content in such products ranges from 1.5–2.5%, contributing both to product functionality and microbial control. Furthermore, cooked sausage needs to be stored under refrigerated conditions to suppress microbial growth and maintain quality. However, the bologna used in this study was freshly prepared for sensory evaluation, and microbiological testing confirmed that the total viable counts were undetectable in all samples observed. SC Biocal had significant effects on all sensory attributes, except for appearance (*p* < 0.05). The highest color, odor, flavor, taste, sandy mouth feel, and over all likeness scores were found for Con 1. For the color, odor, and flavor, Biocal addition resulted in a lower color likeness score for the resulting product. This suggests that the orange-pinkish color is preferred for bologna made from salmon cuts/surimi blends. As the Biocal level was increased, the scores for color and odor decreased, likely due to the increased whiteness and the presence of oxidation products associated with Biocal. The highest texture likeness score was attained for Biocal 8, however, no significant differences between Biocal 6 and 8 were detectable (*p* < 0.05). This agreed with the better texture profiles, BF, GS, and shear force. However, the likeness score decreased when the amount of Biocal was increased to 10% (*p* < 0.05). This reduction could be attributed to the rigid texture of the samples containing a higher concentration of Biocal. Additionally, higher levels of Biocal addition had a detrimental effect on the flavor, taste, and sandy mouthfeel of Biocal-fortified bologna. The flavor and taste likeness scores decreased as Biocal levels increased (*p* < 0.05). Pudtikajorn et al. [39] noted that the remaining protein and residual lipid associated with Biocal could generate fishy nitrogenous compounds and rancidity in a fish tofu product. This result reconfirmed that SC Biocal contains volatile compounds, which likely introduce undesirable flavors affecting the taste of the final product. The lowest sandy mouthfeel likeness score was observed for Biocal 10 (*p* < 0.05), indicating a higher level of sandiness detected. A slight sandy mouthfeel was detected by some panelists. The score for the sandy mouthfeel likeness of Biocal 6 was similarly to that of the control bologna (*p* > 0.05). In a previous study, the SC Biocal particle had a surface mean diameter of 6.86 µm, while the volume mean diameter was measured at 23.65 µm. Yin and Park [47] documented that no sandy mouthfeel was detected in surimi gel incorporated with fish bone powder having a particle size smaller than 150 µm. Similarly, Wijayanti et al. [22] reported that Asian seabass Biocal with a particle size of 16.71 µm caused no sandy mouthfeel when used at levels below 8%. Overall, Biocal 6 was the most preferred Biocal-fortified bologna, as shown by the highest overall likeness score. These results suggested that the addition of SC Biocal at 6% is an appropriate level, with no negative impacts on organoleptic quality. Our findings indicate that the amount of Biocal used to fortify fish-based gel products plays a crucial role, not only in improving gel properties, but also in determining the acceptability of the products.

#### 3.2.5. Microstructure

**Figure 5:**
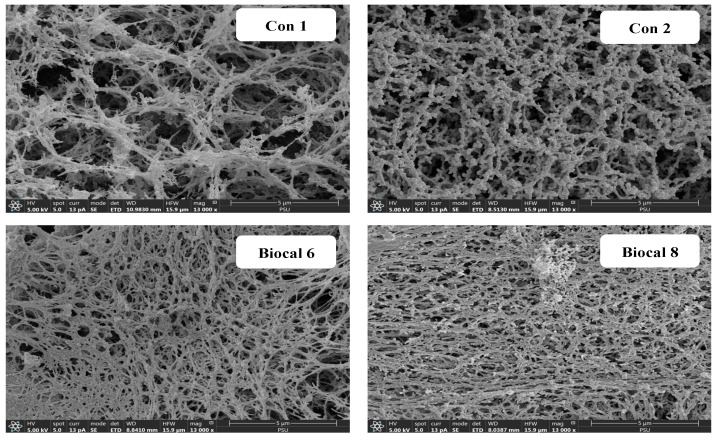
Scanning electron microscopic image of control and selected bologna from salmon cuts/surimi mixed gel containing with 0.05% MTGase and fortified with SC Biocal at levels of 6 and 8%. Magnification: 13,000×.

The microstructure of selected bologna gels made from salmon cut–surimi mixed gels, including (1) Con 1 (without MTGase and Biocal), (2) Con 2 (with MTGase and without Biocal), (3) Biocal 6, and (4) Biocal 8, was visualized by SEM, as shown in Figure 5. The control gel without TGase (Con 1) displayed a more discontinuous network, with larger holes compared to the control gel containing MTGase (Con 2), which aligns with Con 1 having the lowest BF and GS (Figure 2) as well as water-holding capacity (Table 4). In the presence of MTGase, the Biocal-fortified gels (Biocal 6 and Biocal 8) showed a finer and denser structure. Nevertheless, the highest compactness with the highest interconnectivity of proteins was observed in Biocal 8. These observations suggested that the Biocal in the presence of MTGase contributed to network formation through intermolecular ε-(γ-glutamyl) lysine cross-linking and protein aggregation. The results also confirmed that Biocal functioned as a filler within the ordered network structure. This aligns with the findings by Wijayanti et al. [42], who reported that micro-sized Biocal from Asian sea bass not only enhanced protein cross-linking but also filled the gel network. Yin and Park [48] similarly documented that surimi gel containing micro fishbone had a denser structure. Overall, the Biocal played a crucial role in gel strengthening of the bologna from mixed fish, especially when used at an optimal level in combined with MTGase. Although Biocal 8 provided better gel characteristics than Biocal 6, it yielded an adverse effect on the organoleptic properties. Therefore, the fortification of SC at 6% (Biocal 6) is the recommended level for fish bologna development.

## 4. Conclusions

SC bone, a leftover generated during slaughtering, can be converted into high-value-added Biocal. It serves as a natural alternative source of calcium and a potential gel enhancer. SC Biocal is rich in calcium, primarily in the form of hydroxyapatite, and demonstrates high bioavailability and absorption. Furthermore, no harmful metals or pathogens were detected. SC Biocal at a 6% (*w*/*w*) formulation can effectively enhance the gelling properties of bologna made from salmon cut/surimi mixed gel, without adversely affecting the final product. Therefore, SC Biocal shows great potential as a functional food ingredient for surimi-based products, while also increasing their nutritional value, particularly in terms of calcium and phosphorus content. However, bologna is a type of sausage that is highly perishable, with a typical pH close to neutral and high a_w_, indicating a high susceptibility to microbial growth and spoilage. To suppress microbial growth and maintain quality, the finished bologna must be stored in refrigerated conditions.

## Figures and Tables

**Table 1:** The formulation of bologna made from salmon cuts/surimi mixed gel, fortified with SC Biocal at different levels.

Ingredients	Content (g)
Surimi: Salmon cut (70:30)	76.8
Cold water	10.0
Tapioca flour	5.0
Shortening	2.5
Sugar	2.0
Salt	2.0
Ground spices (pepper: ginger: garlic; 2:1:1)	1.0
Carrageenan	0.5
Monosodium glutamate (MSG)	0.2
MTGase (0.05% of fish meat)	0.038
Coloring agent (orange)	0.01

Source: Adapted from Santana et al. [17].

**Table 2:** Yield and physio-chemical and microbial qualities of Biocal from SC bone.

Parameter	Contents
Yield (%)	16.83 ± 0.29
Color	
L*	90.28 ± 0.02
a*	−0.32 ± 0.06
b*	8.25 ± 0.25
Water activity (a_w_)	0.51 ± 0.01
Proximate composition (%)	
Moisture	4.87 ± 0.06
Protein	10.38 ± 0.32
Lipid	2.80 ± 0.09
Ash	69.71 ± 0.14
Mineral component	
Ca (% wt)	26.25
P (% wt)	13.72
Hg (μg/kg)	<5
As (mg/kg)	ND
Pb (mg/kg)	ND
Cd (mg/kg)	ND
Hydroxyproline content (mg/g sample)	2.85 ± 0.26
Crystallinity (%)	69.92
Ca bioavailability (%)	16.10 ± 0.37
Microbial assessment	
Total plate count (CFU/g)	ND
Yeasts and molds (CFU/g)	ND
*Salmonella* spp. (in 25 g)	ND
*Escherichia coli* (MPN/g)	<3
*Staphylococcus aureus* (CFU/g)	ND

ND: Not detectable. Values are given as means ± SD from triplicate determinations.

**Table 3:** Amino acid composition and microbial properties of Biocal from SC bone.

Amino Acid Composition (g/100 g)	Contents
Aspartic acid	0.94
Threonine	0.49
Serine	0.81
Glutamic acid	1.67
Proline	2.95
Glycine	4.40
L-Alanine	2.32
Cystine	ND
Methionine	0.01
Isoleucine	0.61
Leucine	0.61
Tyrosine	0.09
Phenylalanine	0.52
Histidine	ND
Lysine	0.81
L-Arginine	1.59
Tryptophan	ND
Valine	0.43
Hydroxylysine	0.11
Hydroxyproline	2.35
Essential amino acids	4.30
Non-essential amino acids	17.14

ND: Not detectable.

**Table 4:** Texture, color, and sensory properties of bologna from salmon cut/surimi mixed gel containing 0.05% MTGase and fortified with SC Biocal at varying levels (0–10%).

Parameters		Con 1	Con 2	Biocal 2	Biocal 4	Biocal 6	Biocal 8	Biocal 10
TPA								
	Hardness (g)	3600.2 ± 134.5 ^g^	4768.0 ± 226.0 ^bf^	5514.7 ± 175.2 ^e^	6800.7 ± 183.6 ^d^	7717.0 ± 244.8 ^c^	8172.1 ± 164.39 ^b^	9051.2 ± 286.07 ^a^
	Adhesiveness (g × s)	−75.29 ± 1.06 ^a^	−88.79 ± 1.83 ^b^	−106.90 ± 1.78 ^c^	−120.74 ± 4.52 ^d^	−126.06 ± 2.42 ^e^	−133.58 ± 2.22 ^f^	−141.51 ± 3.14 ^g^
	Springiness (cm)	0.71 ± 0.02 ^d^	0.71 ±0.04 ^d^	0.72 ± 0.03 ^d^	0.76 ± 0.01 ^c^	0.80 ± 0.02 ^bc^	0.81 ± 0.00 ^ab^	0.84 ± 0.01 ^a^
	Cohesiveness	0.65 ± 0.13 ^b^	0.65 ± 0.03 ^b^	0.66 ± 0.04 ^b^	0.73 ± 0.03 ^ab^	0.74 ± 0.02 ^ab^	0.76 ± 0.02 ^ab^	0.78 ± 0.04 ^a^
	Gumminess (g)	2333.6 ± 393.5 ^f^	3119.7 ± 162.5 ^e^	3646.0 ± 307.6 ^e^	4976.8 ± 130.5 ^d^	5679.9 ± 309.8 ^c^	6241.5 ± 212.1 ^b^	7049.2 ± 7291.3 ^a^
	Chewiness (kg × cm)	161.69 ± 25.21 ^f^	219.05 ± 20.48 ^e^	258.27 ± 28.03 ^e^	372.97 ± 11.58 ^d^	443.16 ± 26.74 ^c^	498.01 ± 17.47 ^b^	581.48 ± 20.29 ^a^
Shear force (g)		3671.75 ± 61.82^e^	4604.04 ± 111.41 ^e^	5618.47 ± 218.94 ^d^	6737.47 ± 183.15 ^c^	7665.69 ± 263.51 ^b^	8112.12 ± 205.10 ^ab^	9168.43 ± 185.45 ^a^
EMC (%)		2.70 ± 0.09^a^	2.65 ± 0.05 ^a^	2.06 ± 0.11 ^b^	1.95 ± 0.18 ^bc^	1.74 ± 0.11 ^c^	1.70 ± 0.22 ^c^	1.68 ± 0.15 ^c^
Color								
	L*	63.55 ± 0.94 ^d^	64.71 ± 1.09 ^d^	66.37 ± 1.91 ^c^	66.57 ± 0.45 ^bc^	67.05 ± 0.58 ^b^	68.12 ±1.13 ^b^	71.60 ± 1.02 ^a^
	a*	2.11 ± 0.13 ^e^	2.87 ± 0.35 ^d^	3.10 ± 0.41 ^cd^	3.33 ± 0.44 ^bcd^	3.47 ± 0.42 ^bc^	3.87 ± 0.42 ^b^	5.48 ± 0.56 ^a^
	b*	11.19 ± 0.36 ^d^	11.98 ± 0.49 ^c^	11.83 ± 0.48 ^cd^	12.30 ± 0.25 ^c^	13.17 ± 0.61 ^b^	14.04 ± 0.81^a^	14.64 ± 0.41^a^
	Whiteness (%)	61.81 ± 0.97 ^d^	62.62 ± 1.08 ^cd^	64.21 ± 1.84 ^b^	64.22 ± 0.47 ^bc^	64.34 ± 0.59 ^bc^	64.94 ± 1.01 ^bc^	67.58 ± 1.14 ^a^
Likeness score								
	Appearance ^ns^	7.10 ± 1.04	7.17 ± 1.21	7.13 ± 1.36	6.70 ± 1.36	6.90 ± 1.34	6.83 ± 1.53	6.78 ± 1.51
	Color	8.08 ± 1.05 ^a^	7.47 ± 1.13 ^b^	6.93 ± 1.38 ^c^	7.07 ± 1.31 ^bc^	7.33 ± 1.20 ^bc^	7.33 ± 1.24 ^bc^	7.00 ± 1.34 ^bc^
	Texture	6.77 ± 1.33 ^bc^	6.57 ± 1.24 ^c^	6.78 ± 1.44 ^bc^	6.55 ± 1.47 ^c^	7.12 ± 1.22 ^ab^	7.33 ± 1.13 ^a^	6.28 ± 1.58 ^c^
	Odor	7.45 ± 1.13 ^a^	7.5 ± 1.14 ^a^	7.05 ± 1.29 ^ab^	6.6 ± 1.51 ^bc^	6.47 ± 1.61 ^c^	6.45 ± 1.56 ^c^	5.75 ± 1.78 ^d^
	Flavor	7.88 ± 0.92 ^a^	7.33 ± 1.16 ^b^	6.47 ± 1.66 ^cd^	6.32 ± 1.55 ^cd^	6.57 ± 1.44 ^c^	6.20 ± 1.63 ^cd^	5.92 ± 1.69 ^d^
	Taste	7.05 ± 1.33 ^ab^	7.17 ± 1.34 ^ab^	6.95 ± 1.41 ^ab^	6.8 ± 1.47 ^ab^	6.97 ± 0.95 ^ab^	7.25 ± 1.34 ^a^	6.68 ± 1.25 ^b^
	Sandy mouth feel	7.19 ± 0.91 ^a^	7.14 ± 1.17 ^a^	7.07 ± 1.26 ^a^	7.09 ± 1.18 ^a^	6.74 ± 1.11 ^a^	5.52 ± 1.50 ^b^	5.05 ± 1.57 ^b^
	Overall	7.73 ± 1.33 ^a^	7.48 ± 1.34 ^a^	7.40 ± 0.86 ^ab^	7.47 ± 1.18 ^a^	7.42 ± 0.95 ^ab^	7.13 ± 1.55 ^bc^	5.78 ± 1.61 ^d^

Mean ± S.D (n = 3). ^ns^ denotes not significant. Different lowercase superscripts within the same column indicate the significant differences (*p* < 0.05). Con 1: control bologna gel without MTGase and Biocal; on 2: control bologna gel containing MTGase without Biocal added; Biocal 2, Biocal 4, Biocal 6, Biocal 8, and Biocal 10: Biocal-fortified bologna gel at 2, 4, 6, 8, 10% (*w*/*w*), respectively, and incorporated with MTGase.

## Data Availability

The original contributions presented in the study are included in the article, further inquiries can be directed to the corresponding author.
